# Antimicrobial activity of cleanser tablets against *S. mutans* and *C. albicans* on different denture base materials

**DOI:** 10.1186/s12903-024-04403-6

**Published:** 2024-05-29

**Authors:** Şükriye Ece Geduk, Gaye Sağlam, Füsun Cömert, Gediz Geduk

**Affiliations:** 1 Faculty of Dentistry, Department of Prosthodontics, Zonguldak Bulent Ecevit University, Kozlu, Zonguldak, Turkey; 2 Faculty of Medicine, Department of Microbiology, Zonguldak Bulent Ecevit University, Zonguldak, Turkey; 3 Faculty of Dentistry, Department of Dentomaxillofacial Radiology, Zonguldak Bulent Ecevit University, Zonguldak, Turkey

**Keywords:** Denture base materials, 3D printed resin, Polyamide, Denture cleanser tablets, *S. mutans*, *C. Albicans*

## Abstract

**Background:**

In this study, the antimicrobial activity of three different cleanser tablets on *S. mutans* and *C. albicans* adhesion to PMMA, polyamide and 3D printed resin was investigated.

**Methods:**

40 samples were prepared for PMMA (SR Triplex Hot), polyamide (Deflex) and 3D printed resin (PowerResins Denture) materials and divided into four subgroups for cleansers (Aktident™, Protefix™, Corega™ tablets and distilled water) (*n* = 5). After the surface preparations were completed, the samples were immersed separately in tubes containing the prepared microorganism suspension and incubated at 37˚C for 24 h. After the incubation, the samples were kept in the cleanser solutions. The samples were then transferred to sterile saline tubes. All the tubes were vortexed and 10 µl was taken from each of them. Sheep blood agar was inoculated for colony counting. The inoculated plates were incubated for 48 h for *S. mutans* and 24 h for *C. albicans*. After incubation, colonies observed on all plates were counted. Statistical analyses were done with three-way ANOVA and Tukey’s multiple comparison test.

**Results:**

Polyamide material registered the highest colony count of *S. mutans*, whereas PMMA registered the lowest. Significant differences in *S. mutans* adherence (*p* = 0.002) were found between the three denture base materials, but no such difference in *C. albicans* adherence (*p* = 0.221) was identified between the specimens. All three cleanser tablets eliminated 98% of *S. mutans* from all the material groups. In all these groups, as well, the antifungal effect of Corega™ on *C. albicans* was significantly higher than those of the other two cleanser tablets.

**Conclusions:**

According to the study’s results, it may be better to pay attention to surface smoothness when using polyamide material to prevent microorganism retention. Cleanser tablets are clinically recommended to help maintain hygiene in removable denture users, especially Corega tablets that are more effective on *C. albicans*.

## Background

Many different base materials have been used to fabricate denture bases. The most commonly used material is polymethylmethacrylate (PMMA), but polyamides, acrylic resin derivatives, and metal alloys are also favored alternatives. PMMA remains superior to these because of its ease of manipulation, biocompatibility, dimensional stability, and economic properties [1, 2].

Despite the advantages of PMMA, however, it also suffers from certain disadvantages, such as a porous surface. Interdental spaces, the metal retainers for removable prostheses, and the indentations and protrusions created during the modeling of prostheses are conducive to microorganism retention. The scratches and microporosities that may occur during the polymerization and polishing of prostheses cause plaque to accumulate and bacterial and fungal colonies to proliferate [3, 4]. In addition to the disadvantages of PMMA, allergic reactions and the increasing aesthetic expectations of patients have prompted a search for alternative base materials. Examples include polyamides, which are thermoplastic polymers formed by the condensation reaction of a diamine and dibasic acid. These materials have high elasticity, sufficient strength, low amount of residual monomer, and superior aesthetic properties [5, 6]. The widespread use of acrylic resins and advances in material technology allow the use of different production techniques, such as subtractive and additive manufacturing [7]. Additive manufacturing, also known as three-dimensional (3D) printing, is a rapidly developing technique for creating objects by layering, used in many fields for dentistry, such as surgical guides, temporary restorations, and resin models. This technology can also contribute to the reduction of technical errors and the production of complex designs. Among its limitations are the insufficient resolution of current printers and shortcomings related to repetitive production [8–10].

Among oral flora, *Streptococcus mutans* is the microorganism that initially attaches to the surfaces of prostheses. The attachment of *S. mutans* to flat surfaces is explained by electrostatic forces [11, 12]. When *S. mutans* colonize in the oral flora, they provide not only an adhesion surface but also lactate, which can supply a carbon source on which *Candida albicans* cells can grow [13]. *C. albicans* is the most important etiologic cause for denture stomatitis. The literature reported that *C. albicans* is the most commonly proliferating microorganism in removable dentures (65%), while *S. mutans* and *S. aureus* are found in 53.3% and 34.4% of such prostheses, respectively [14–16].

Effective cleaning of dentures is necessary to prevent oral infection and systemic spread [17, 18]. Cleaning can be achieved through mechanical or chemical approaches or a combination of these, but the most preferred and economical method is brushing. The problem is that elderly patients cannot carry out effective brushing because of their poor manipulation skills and diminished vision [19, 20]. Therefore, the use of cleaning solutions that contribute to prosthetic hygiene by sanitizing hard-to-clean pores and spaces has become widespread. The most popular chemical cleaners are alkaline peroxides (sodium perborate, potassium monopersulfate), alkaline hypochlorites (sodium hypochlorite, trisodium phosphate), disinfectants (chlorhexidine, glutaraldehyde), diluted acids (hydrochloric acid, phosphoric acid), and enzymes [21–23]. Alkaline peroxide agents are available in powder form or as effervescent tablets. When alkaline peroxide is dissolved in water, the perborate produced reacts with the water, and a peroxide solution is formed, thereby releasing oxygen. This oxygen release plays a role in the micromechanical removal of attachments on prostheses. If used regularly, these cleaners can effectively remove residues [22, 24].

Studies have examined the effects of cleaning agents on the microbial uptake of acrylic base materials [19, 25–27], but the data is limited on which agent reduce the uptake of *C. albicans* and *S. mutans* in PMMA, polyamide, and 3D printing resins. The current research investigated how different cleanser tablets affect microorganism retention in PMMA, polyamide, and 3D-printed acrylic resin base materials. The investigation was guided by the following null hypotheses:

1. Differences in materials do not change the uptake of microorganisms.
2. There is no difference between the cleaning efficacy of various cleanser tablets.

## Methods

### Preparation of samples

**Fig. 1:**
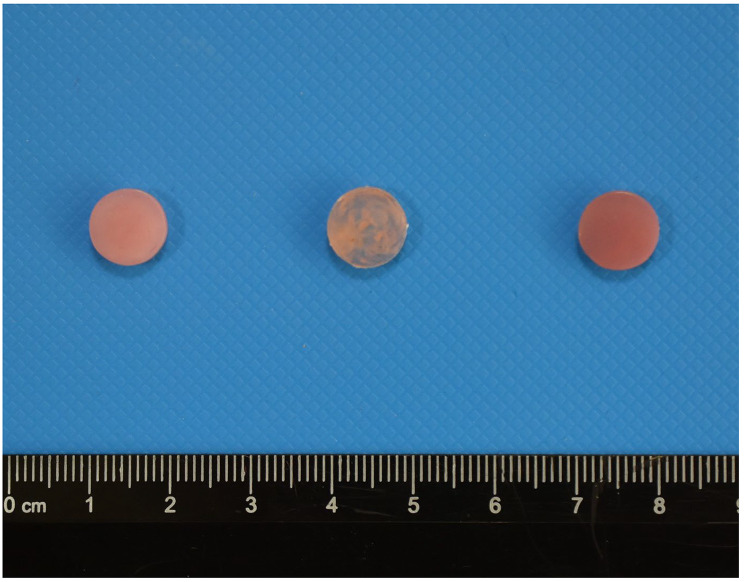
Samples prepared for use in the study (respectively PMMA, polyamide, 3D Print)

Three base materials were used in the study: PMMA, polyamide, and 3D printed acrylic resin (Table 1). The minimum sample size to be included in the study was calculated as 4.85 ~ 5 for each group with a large effect size (f = 0.40), a type 1 error value of 0.05 and a power value of 0.90, for a total of 120 samples. In order to calculate the sample size, G-Power ver. 3.1.9.4 program was used [35, 36]. For each group of materials, 40 disc-shaped specimens (Ø10 × 3 mm ) were prepared (Fig. 1). PMMA samples were produced by using conventional heat polymerization technique whereas polyamide fabricated by injection-moulding. 3D printed samples were designed as a virtual Ø10 × 3 mm round disk and saved as a standard tessellation language file using a software program (Mashmixer, Autodesk). The design was crafted onto a biocompatible acrylic resin (PowerResins Denture™, Istanbul, Turkey) at a structural orientation of 90° using a digital light processing-based 3D printer (Dentafab Sega, 3Bfab Technology Company, Istanbul, Turkey). The specimens were soaked in isopropyl alcohol for one minute, then air-dried and postpolymerized in an ultraviolet device (Medifive, Twin Cure, Korea) at 405 nm for 10 min. To ensure standardization, the surfaces of all the samples were ground with 800-, 1000-, and 1200-grit silicon carbide abrasives (Gripo 2 V, Metkon, Bursa, Turkey) at 100 rpm under water cooling. The roughness of the standardized surfaces was checked using a contact profilometer (Surtronic 25, Taylor Hobson, Leicester, UK) with a measuring length of 4 mm and a speed of 1 mm/sec. Surface roughness values are shown in Table 2. For each material type, samples were randomly selected and divided into two subgroups to be tested using *S. mutans* and *C. albicans*.

**Table 1:** Denture base materials used in the study and their contents

Material	Manufacturer	Content	Production Method
**SR Triplex Hot™**	Ivoclar Vivadent Inc. Schaan. Liechtenstein	PMMA	Muflling
**Deflex™**	Nuxen S.R.L. Buenos Aires. Argentina	Polyamide	Injection
**PowerResins Denture™**	Istanbul. Turkey	3D Resin	3D Printed

**Table 2:** Surface roughness values of the denture base materials

Material	Mean ± sd
PMMA	0.30 ± 0.05 μm
Polyamide	0.34 ± 0.02 μm
3D Printed	0.28 ± 0.03 μm

### Preparation of cleanser tablet solutions

To examine antimicrobial efficacy, four groups were formed: three that were cleaned using alkaline peroxide cleanser tablets and a control group cleaned by immersion in distilled water (*n* = 5). The tablets were reprepared daily with distilled water according to the manufacturers’ instructions. The manufacturers and usage details of the tablets are presented in Table 3.

**Table 3:** Cleanser tablets used in the study and their ingredients

Cleanser Tablet	Manufacturer	Content	Preparation advice	Contact Time
**Corega™**	GlaxoSmithKline Healthcare. Istanbul. Turkey	Sodium bicarbonate. citric acid. potassium monopersulfate. sodium carbonate. sodium carbonate peroxide. TAED. sodium benzoate. PEG-180. sodium lauryl sulfoacetate. sodium perborate monohydrate VP/VA copolymer. flavor. subtilisin	1 tablet with 200 ml water	15 min
**Aktident™**	Aktif Dis Ticaret. Istanbul	Potassium caroate. sodium bicarbonate. citric acid. sodium carbonate. sodium lauryl sulfate. sodium lauryl sulfoacetate. flavor	1 tablet with 200 ml water	15 min
**Protefix™**	Quiesser Pharma. Flensburg. Germany	Sodium bicarbonate. Potassium carbonate. Sodium perborate. Citric acid. Sodium lauryl sulfate. Flavor	1 tablet with 100 ml water	10 min

### Microorganism experiments

Surface preparations were completed, and the samples were sterilized with ethylene oxide. Bacterial suspensions containing *S. mutans* (obtained from the National Culture Collection of the Public Health Institution of Turkey) and *C. albicans* (patient isolate) strains were adjusted to a turbidity of 0.5 McFarland (10 for colony forming units (CFUs)/ml) using tryptic soy broth (1 ml) and brain–heart infusion broth (1 ml), respectively. All specimens whose surfaces were to be tested were immersed in tubes containing the prepared microorganism suspensions and incubated at 37 °C for 24 h. After incubation, all the samples were transferred into sterile tubes. Sterile saline (1 ml) was added to the tubes containing the control specimens, whereas the solutions (1 ml) prepared according to the manufacturers’ instructions were added to the tubes where the three other groups of specimens were kept (Table 3). The temperature at the surfaces of the prepared tubes was kept at 37 °C using cleaners. The samples were then transferred to tubes containing 500 µl of sterile saline. Each tube was vortexed for 30 s to allow the remaining microorganisms to flow into the saline. After vortexing, 10 µl of the solution in each tube was spread onto the surface of sheep blood agar (Oxoid™ Blood Agar Base, CM0055, Thermofisher Scientific, Denmark) plates for inoculation and colony counting. The plates inoculated with *S. mutans* were incubated for 48 h under a 5% CO atmosphere, whereas those inoculated with *C. albicans* were incubated for 24 h under normal atmospheric conditions. After incubation, the colonies observed on all the plates were counted using a Quebec Colony Counter (American Optical Corp., Buffalo, NY, USA) and on the basis of the classical formula for CFUs. The values obtained were multiplied by 100 to calculate CFU/ml.

### Statistical analyzes

Data analyses were performed using JAMOVI (v. 2.0.0.0) and IBM SPSS (v. 23.0). Descriptive statistics are presented as mean ± standard deviation (mean ± SD). The normality of the data was assessed using the Kolmogorov-Smirnov test. As the data were normally distributed, they were analysed using three-way factorial ANOVA. In cases wherein differences were detected in the analysis, Tukey’s multiple comparison test was conducted to investigate the groups that caused such dissimilarities. A *p* < 0.05 was considered indicative of statistical significance.

## Results

**Fig. 2:**
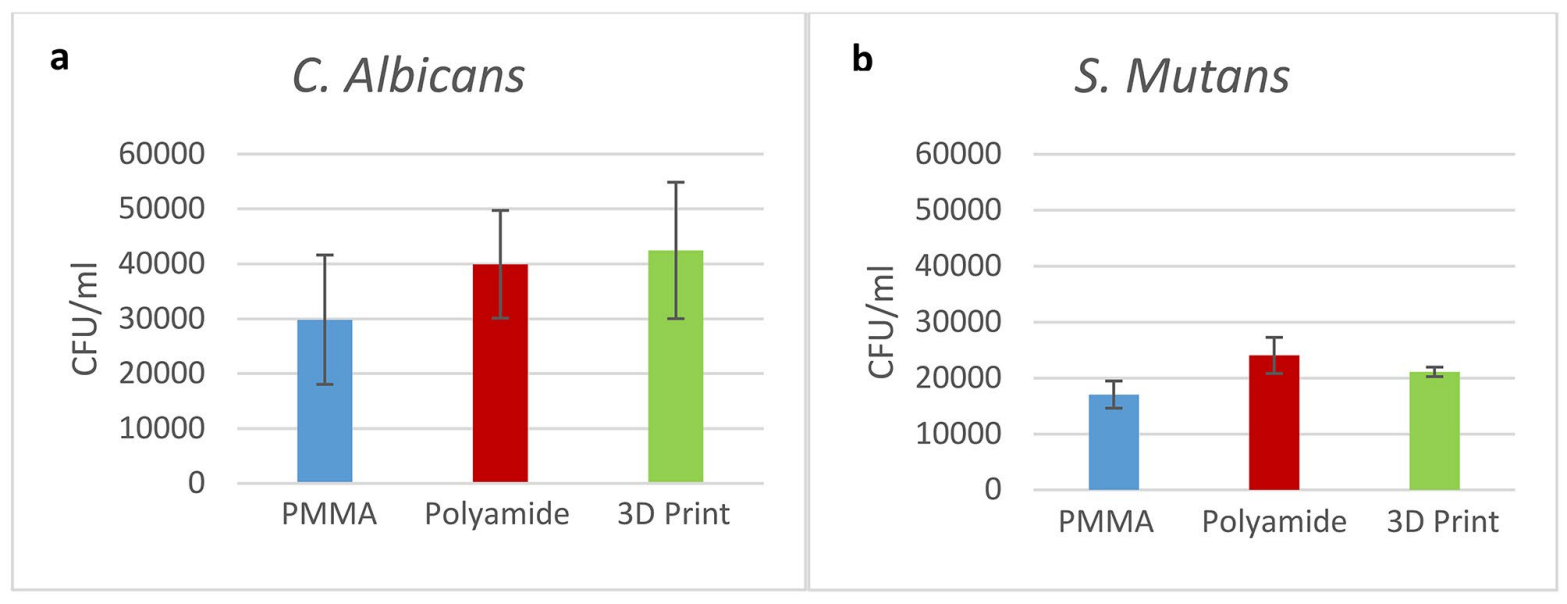
Mean colony counts of microorganisms in 1 mL of control groups **(a)***C. Albicans***(b)***S. Mutans*

The average counts of *S. mutans* and *C. albicans* colonies in the control group are shown in Fig. 2. Polyamide material registered the highest colony count of *S. mutans*, whereas PMMA registered the lowest. Significant differences in *S. mutans* adherence (*p* = 0.002) were found between the three denture base materials, but no such difference in *C. albicans* adherence (*p* = 0.221) was identified between the specimens.

The descriptive statistics of the efficiency with which different microorganisms were cleaned and the efficiency of cleanser tablets across different acrylic resins are provided in Table 4. The three-way ANOVA uncovered significant differences in microbial adhesion between material types (< 0.0001), tablet types (< 0.0001), microorganism types (< 0.0001), interactions between microorganism–tablet factors (< 0.0001), and interactions between microorganism–material factors (0.006). No significant difference was found between the interactions of material, tablet, and microorganism types (*p* = 0.087) (Table 5). The control group (distilled water immersion) exhibited higher *S. mutans* and *C. albicans* adhesion than the tablet-treated groups, and this difference was significant. However, the *C. albicans* specimens cleaned with Corega™ showed significantly lower colony counts than those treated using Aktident™ and Protefix™, as determined by Tukey’s HSD test (Table 6). No significant difference in *S. mutans* colony count was found between the tablet-treated PMMA, polyamide, and 3D-printed specimens. Lower *C. albicans* adherence was found on the PMMA resin specimens, and this significantly differed from adherence in both the polyamide and 3D-printed samples after treatment with the cleanser tablets (Table 7).

**Table 4:** Descriptive statistics of the cleaning efficiency measurements of different microorganisms and cleanser tablets on different acrylic resins

Microorganism	Cleanser Tablet		Material		
			PMMA	Polyamide	3D resin
S. Mutans	Aktident™	Mean ± SD	140.0 ± 313.0	12.0 ± 21.7	0.0 ± 0.0
	Protefix™	Mean ± SD	4.0 ± 5.5	188.0 ± 201.9	62.0 ± 138.6
	Corega™	Mean ± SD	0.0 ± 0.0	0.0 ± 0.0	0.0 ± 0.0
	Control	Mean ± SD	17032.0 ± 2425.4	24039.2 ± 3229.5	21093.6 ± 852.6
C. Albicans	Aktident™	Mean ± SD	17064.0 ± 926.8	36352.0 ± 6363.3	24530.0 ± 9274.1
	Protefix™	Mean ± SD	23912.0 ± 8342.8	34520.0 ± 3537.2	28480.0 ± 12084.4
	Corega™	Mean ± SD	124.0 ± 203.2	2.0 ± 4.5	576.6 ± 776.4
	Control	Mean ± SD	29824.0 ± 11783.4	39920.0 ± 9815.9	42440.0 ± 12430.9

*Mean: Average. SD: Standard deviation*

**Table 5:** Investigation of the effect of different microorganisms and cleanser tablets on the antibacterial activity of different acrylic resins

Source of Change	Degrees of Freedom	Adjusted Sum of Squares	Adjusted Mean Squares	F-value	*p*-value
Microorganism	1	9645784210.0	9645784210.0	303.4	**< 0.0001**
Cleanser tablet	3	12617970000.0	4205990000.0	132.3	**< 0.0001**
Material	2	701491212.5	350745606.3	11.0	**< 0.0001**
Microorganism*Cleanser tablet	3	3740823636.0	1246941212.0	39.2	**< 0.0001**
Microorganism*Material	2	345620507.8	172810253.9	5.4	**0.006**
Cleanser tablet *Material	6	388591246.9	64765207.8	2.0	0.068
Microorganism*Cleanser tablet*Material	6	363900148.9	60650024.8	1.9	0.087
Error	96	3052309383.0	31794889.4	---	---
**Total**	119	30856490340.0	---	---	---

^22^S= 5638.696. *R* = 90.1%. Adj-*R* = 87.7

**Table 6:** Descriptive Statistics and Multiple Comparison Results for Microorganism and Cleanser Tablet Interaction

Microorganism		Cleanser Tablet			
		Aktident™	Protefix™	Corega™	Control
S. Mutans	Mean ± SD	50.7 ± 180.1^A^	84.7 ± 153.2^A^	0.0 ± 0.0^A.a^	20721.6 ± 3702.8^B^
C. Albicans	Mean ± SD	25982.0 ± 10195.7^A^	28970.7 ± 9241.7^A^	234.2 ± 499.5^B.a^	37394.7 ± 11966.2^C^

*Mean: Average. SD: Standard deviation*

*There is no statistically significant difference (*p* > 0.05) between cleanser tablets with a common capital letter in the same microorganism type.

*There is no statistically significant difference between microorganism species with a common lower case letter in the same cleanser tablet type (*p* > 0.05).

**Table 7:** Descriptive Statistics and Multiple Comparison Results for Microorganism and Material Interaction:

Microorganism		Material		
		PMMA	Polyamide	3D Resin
S. Mutans	Mean ± SD	4294.0 ± 7628.5^A^	6059.8 ± 10753.3^A^	5288.9 ± 9370.3^A^
C. Albicans	Mean ± SD	17731.0 ± 13202.6^A^	27698.5 ± 17451.9^B^	24006.7 ± 17909.0^B^

*Mean: Average. SD: Standard deviation*

* There is no statistically significant difference between materials with a common capital letter in the same microorganism type (*p* > 0.05).

The results on the cleaning efficiency (in percentage) of the tablets for the acrylic resins are listed in Table 8. All three cleanser tablets eliminated 98% of *S. mutans* from all the material groups. In all these groups, as well, the antifungal effect of Corega™ on *C. albicans* was significantly higher than those of the other two cleanser tablets (Aktident™ and Protefix™).

**Table 8:** Antimicrobial activity* of different cleanser tablets on different acrylic resins

	S. Mutans*			C. Albicans*		
	Aktident	Protefix	Corega	Aktident	Protefix	Corega
PMMA	99.18	99.98	100.00	42.78	19.82	99.58
Polyamide	99.03	99.97	100.00	8.94	13.53	99.99
3D resin	98.83	99.97	100.00	42.20	32.89	98.64

*Antimicrobial activity: Calculated according to the formula 100*(Cn - Tn)/ Cn. Cn: Number of colonies in the control group. Tn: Number of colonies in the tablet group

## Discussion

Different production techniques affect the surface structures of materials and the attachment of microorganisms [28–31]. This study examined *C. albicans* and *S. mutans* adhesion on removable prosthetic materials fabricated through varying techniques and determined the effectiveness of denture cleanser tablets in removing these microorganisms from material surfaces. The null hypotheses were partially rejected. The results showed that *C. albicans* viability was influenced by the brand of cleanser tablet and the type of resin used.

In this study, *C. albicans* showed more adhesion to the base materials than *S. mutans*. This may be due to the differences in the adhesion mechanism between the two microorganisms. While *S. mutans* adheres to flat surfaces using electrostatic forces [12], *C. albicans* adheres in two stages. In the first stage, the microorganism reversibly adheres to the surface and in the second stage, colonisation and tighter attachment occurs [32]. In agreement with our results, Ozel et al. [33] investigated the retention of *S. mutans* and *C. albicans* on temporary crown materials and reported that Candida albicans was more prevalent in all material groups.

Polyamide resins generally exhibit a rougher structure than PMMA surfaces [25, 34]. To prevent effects from these differences, all the sample groups in this study were standardized through typical surface modification procedures, and their initial surfaces were measured using a profilometer (PMMA: 0.30 ± 0.05 μm, Polyamide:0.34 ± 0.02 μm, 3D: 0.28 ± 0.03 μm). Nevertheless, the highest *S. mutans* uptake in the control group was observed in the polyamide resins, followed by the 3D-printed specimens.

It was observed that the lowest retention of both microorganisms was in the PMMA group. A previous study on this topic also found that the retention of *C. albicans* was lower in PMMA than in polyamide, and this difference was explained by the fact that the residual monomer in PMMA inhibits adhesion by reducing the surface energy [35]. Meirowitz et al. [36] also reported in their study that 3D printed resins may play a predisposing role in denture stomatitis due to a higher colonisation of *C. albicans* compared to heat cured resins. The chemical composition of the 3D printed and conventional PMMA resins are almost similar, but the fabrication techniques reveals different. Several studies have reported inconsistent results with regarding the adhesion of *C. albicans* to 3D-printed dentures. The surface properties of the 3D resin can be influenced by factors such as the type of printer, layer thickness and structure angle, and printing orientation [37–39]. Koujan et al. [40] explored the adhesion of *C. albicans* to acrylic resins produced by different techniques (heat polymerization, CAD/CAM milling, and 3D printing) and found that microorganism adhesion is highest in 3D-printed acrylic resin. Another study inquired into the effects of acrylic production methods on *C. albicans* uptake and biofilm formation. The authors reported that the surface topography of 3D-printed resins increased microorganism uptake and that patients using prostheses made with this material should be closely monitored for oral hygiene [41]. In the present research, no statistical difference was found between the amount of colonization of *C. albicans* on different base materials, but the numerical data showed that these microorganisms were most prevalent in the 3D-printed resins. This result may be due to the surface properties of the layers in resins fabricated via 3D printing. Many authors have also reported that the dimpled surfaces formed by the connection between such layers irreversibly stimulate the settlement of microorganisms [42, 43].

Alkaline peroxide effervescent tablets usually contain sodium perborate and sodium bicarbonate. The sodium perborate dissociates from the alkaline peroxide solution formed when the tablets are dissolved in water. The peroxide solution releases oxygen and mechanically removes the debris; also produces oxygen free radicals with antimicrobial activity and enzymes that degrade biofilm proteins [44]. The actident cleanser tablets used in this study contained sodium bicarbonate, while Protefix and Corega tablets contained both sodium bicarbonate and sodium perborate and were used at different immersion times.

Most studies have been conducted on *C. albicans*, and no research has been found on the effects of cleanser tablets on *S. mutans* uptake on polyamide resins [25, 45]. In this study, results indicated that all the effervescent tablets showed strong antimicrobial activity against *S. mutans* colonization because of their superficial location on biofilm in the three acrylic resin groups, but they, except for Corega™, achieved insufficient effects against *C. albicans*. These results are compatible with the literature. For example, Souza et al.’s [46] study on the cleaning efficacy of propolis solution as well as saline and alkaline peroxide solutions on PMMA acrylic resins showed that Corega™ tablets containing alkaline peroxide are significantly effective against *S. mutans* and *C. albicans*. Another in vitro study on the efficacy of different alkaline peroxide tablets on some biofilms showed that Corega™ tablets effectively reduce the viability of both *S. mutans* and *C. albicans* biofilms [47]. Meanwhile, Andrade et al. [44] used Corega™ tablets and set the immersion time to 5 min to delve into the efficacy of effervescent tablets and ultrasonic cleaners against *C. albicans* and *S. mutans* in prosthesis biofilms. The authors found that the tablets significantly reduce *S. mutans* colonization but are inadequate against *C. albicans*. In our study, the holding times recommended by the manufacturers of the cleanser tablets (Corega™ = 15 min, Aktident™ = 15 min, and Protefix™ = 10 min) were used. The difference between this study and that of Andrade et al. in terms of the functioning of Corega™ may be related to the immersion time of the tablets. Similarly, the results derived by Volchkova et al. [48] differ from those of our research. The authors evaluated the effectiveness of cleansing soap and Protefix™ tablets on microorganisms in removable prostheses and noted that the latter satisfactorily reduces the amount of *C. albicans* on the materials under an immersion time of 15 min. Drake et al. [49] examined the effectiveness of antibacterial alkaline peroxide tablets on *C. albicans* and *S. mutans*. The authors similarly showed that whereas *S. mutans* colonization is largely eliminated, no significant reduction in colony occurs with respect to *C. albicans*.

The study by Hayran et al. [50] reported that the polarity of the resins may influence the anticandidal activity of denture cleanser tablets and that Corega tablets can be applied to all resin types. Although the polarities of the different base resins were not evaluated in our study, there are similarities with the relevant study in terms of the study groups (heat-cured PMMA, thermoplastic resin).

This in vitro study has some limitations. Under in vivo conditions, the antimicrobial properties of saliva may help to wash dentures and reduce microbial adhesion. Also, mechanical cleaning procedures and unpolished areas in contact with oral tissues can increase the retention of microorganisms. The parameters tested should be evaluated in an in vivo model to clearly understand the behavior of these materials and denture cleansers. Further studies are needed to investigate the activity and synergistic behavior of microorganisms in the complex biofilm environment.

## Conclusions

These findings suggest that polyamide materials should have a smooth surface to avoid microbial retention. Cleanser tablets, especially Corega™ which are more effective against *C. albicans*, are clinically recommended to maintain hygiene in users of removable dentures. Further studies should focus on material parameters related to microbial growth.

## Data Availability

The datasets used and/or analysed during the current study are available from the corresponding author on reasonable request.

## Abbreviations

PMMA: Poly Methyl Methacrylate
CAD-CAM: Computer aided design-Computer aided manufacturing
3D: Three dimensional
